# Irritability in Adult Depression and Anxiety: A Clinically Useful Signal Amid an Imprecise Construct

**DOI:** 10.1177/07067437261490170

**Published:** 2026-10-14

**Authors:** Michael Uebel

**Affiliations:** 1 Steve Hicks School of Social Work, Office of the Associate Dean for Research, 12330 The University of Texas at Austin, Austin, TX, USA

**Keywords:** depressive disorders, anxiety, irritability, diagnosis, clinical assessment

## Abstract

A brief consideration of the conceptual and diagnostic tidiness of irritability associated with depression and anxiety in adults. Despite evidence for its conceptual untidiness, there are practical issues that the clinician may consider when diagnosing and treating depression or anxiety.

A 34-year-old man presents to his family physician reporting poor sleep and a “short fuse.” He denies sadness. His partner, present at his request, describes weeks of snapping over trivia, withdrawal, and uncharacteristic anger. He works long hours and drinks more than he used to. He does not think he is depressed. He is wrong, and his irritability is the clue.

Irritability is among the most common and least examined features of mood and anxiety disorders in adults. It is near ubiquitous in mood disorders, yet it remains poorly understood and under-researched in adults specifically.^1^ Roughly half of adults in a depressive episode report irritability for much of that episode, and its presence marks greater symptom severity, earlier onset, and a more chronic course.^2^ For the clinician, the practical question is not whether irritability is conceptually tidy, for the simple reason that it is not. The question is what to do with it, and here the evidence is more helpful than its reputation suggests.

## A Construct Still in Search of a Definition

The literature offers many definitions and measures of irritability, most overlapping with anger and aggression.^3^ A concept analysis converges on a practicable core: a lowered threshold for anger and negative affect, reduced control over emotion, provoked by frustration or physiological states such as poor sleep, hunger, and pain, and often experienced as disproportionate to its trigger.^3^ Crucially, irritability is a subjective experience that need not produce observable aggression, which distinguishes it from the behavioural outbursts that dominate paediatric conceptualizations.^1^ Adults can introspect on this state, which makes self-report instruments such as the Irritability Questionnaire usable in practice, alongside brief embedded measures.^4^ The Irritability Questionnaire remains one option, but newer instruments have since been validated in an Italian adolescent and adult population: the Affective Reactivity Index (ARI) and the Born-Steiner Irritability Scale (BSIS) both show good internal consistency and excellent test–retest reliability, and their brevity makes them practical for baseline and repeat administration. Both scales were developed in English, with the ARI originally for children and adolescents, and the BSIS created for the assessment of irritability in women with mood disorders related to the menstrual cycle, pregnancy, or menopause. Notably, the BSIS has also been used to assess irritability in the general population and related to other health conditions. Because the ARI rates the past week and the BSIS a longer interval (the past 6 months), the 2 together suit serial monitoring of change across different timeframes.^5^

For diagnosis, attention to developmental differences is important. The DSM counts irritability as a depressive symptom in children and adolescents but lists it under mania for adults, dividing the construct arbitrarily by age and mood polarity.^1^ The consequence is a clinical blind spot: an adult presenting with prominent irritability and depressed mood may not map cleanly onto the depressive criteria a clinician is scanning for.

## Why Irritability Correlates With Depression and Anxiety

The association is not incidental. In youth, longitudinal and genetically informed studies show that irritability specifically predicts later depression and anxiety, and that its overlap with depression is largely explained by shared genetic factors.^6^ Recent genome-wide work in adults points the same way: depressed adults with and without prominent irritability are genetically almost identical and share the same links to anxiety and neuroticism, suggesting irritable depression is not a separate illness but the same condition in a different guise.^7^ Irritability and anxiety appear to share neurobiological origins, plausibly representing approach and avoidance sides of a common threat-response system.^8^ For the clinician, the takeaway is that irritability in a depressed or anxious adult is part of the illness, not a personality flaw or a comorbid nuisance to be managed separately.

## Irritability at Intake: More Than a Distraction

The strongest case for routinely assessing irritability is prognostic. In a large, replicated analysis of treatment-seeking outpatients with major depression, early reduction in irritability over the first four weeks of antidepressant treatment predicted higher likelihood of remission and lower likelihood of no meaningful benefit, independent of change in depression severity itself.^8^ A one-standard-deviation greater reduction in irritability predicted a 1.73-fold higher likelihood of remission.^8^ Improvement in irritability appeared as early as week one and was not fully accounted for by improvement in core depressive symptoms.^8^ This means irritability carries information that standard depression scales miss, and that tracking it offers an early, actionable read on whether to continue or modify a treatment.

Irritability may also flag depression that would otherwise be missed. Men are diagnosed with major depression at roughly half the rate of women, and there is longstanding concern that some men express depression through externalizing features, including anger.^9^ Empirical work supports trait anger as a predictor of depressive symptoms in men.^9^ The stakes are significant since men account for roughly three-quarters of suicides yet are diagnosed with depression far less often than women, a gap that an anger-dominated presentation may help explain.^9^ The man in the vignette, who denies sadness but presents irritable, withdrawn, and drinking more, fits this pattern. Treating his irritability as an entry point rather than a distraction may be what gets him diagnosed and treated.

## Management Guidance

There is no agent that specifically and reliably reduces irritability, and most evidence derives from secondary analyses.^1^ Several practical principles follow from the literature. First, assess irritability at the outset and re-measure it early, because its trajectory in the first weeks is informative.^8^ Second, treat the underlying disorder rather than the irritability in isolation; serotonergic antidepressants reduce irritability in depression and premenstrual dysphoric disorder, and the presence of irritability may itself predict responsiveness to specific agents.^1,8^ Third, attend to modifiable physiological contributors such as sleep loss, which lower the threshold for irritability and are correctable.^3^ Irritability in bipolar disorder carries its own treatment considerations and is beyond the scope of this piece. Psychological strategies, including cognitive behavioural approaches that target hostile appraisals and frustration tolerance, are reasonable adjuncts.^1^

The construct will remain imperfect until the subjective experience of irritability in adults is better characterized.^1^ Clinicians need not wait. Irritability is impairing, it is prevalent, it predicts course and treatment response, and it can reveal depression that hides behind a denial of sadness.

## Clinical Pearls

- Ask about irritability routinely in adults with depressed or anxious mood, and re-measure it within the first few weeks; early improvement predicts remission independent of change in core depressive symptoms.
- Treat irritability as part of the illness rather than a separate problem, targeting the underlying disorder while correcting modifiable physiological drivers such as sleep loss.
- Consider prominent irritability, anger, and externalizing behaviour as a possible presentation of depression in men who deny sadness, which may reduce missed diagnoses.
